# Clinical Use of Short-Course and Low-Dose Corticosteroids in Patients With Non-severe COVID-19 During Pneumonia Progression

**DOI:** 10.3389/fpubh.2020.00355

**Published:** 2020-07-03

**Authors:** Zhiliang Hu, Yanling Lv, Chuanjun Xu, Wenkui Sun, Wei Chen, Zhihang Peng, Chen Chen, Xiang Cui, Damin Jiao, Cong Cheng, Yun Chi, Hongxia Wei, Chunmei Hu, Yi Zeng, Xia Zhang, Yongxiang Yi

**Affiliations:** ^1^Nanjing Infectious Disease Center, The Second Hospital of Nanjing, Nanjing University of Chinese Medicine, Nanjing, China; ^2^Center for Global Health, School of Public Health, Nanjing Medical University, Nanjing, China; ^3^Department of Respiratory Medicine, The Second Hospital of Nanjing, Nanjing University of Chinese Medicine, Nanjing, China; ^4^Department of Radiology, The Second Hospital of Nanjing, Nanjing University of Chinese Medicine, Nanjing, China; ^5^Department of Respiratory & Critical Care Medicine, The First Affiliated Hospital of Nanjing Medical University, Nanjing, China; ^6^Department of Clinical Research Center, The Second Hospital of Nanjing, Nanjing University of Chinese Medicine, Nanjing, China; ^7^School of Public Health, Nanjing Medical University, Nanjing, China; ^8^Department of Tuberculosis, The Second Hospital of Nanjing, Nanjing University of Chinese Medicine, Nanjing, China

**Keywords:** COVID-19, SARS-CoV-2, corticosteroids, virus shedding, short-course, low-dose, intravenous immunoglobulin

## Abstract

**Background:** The emerging coronavirus disease 2019 (COVID-19) has become a serious public health concern with a high number of fatalities. It is unclear whether corticosteroids could be a candidate for an early intervention strategy for patients with COVID-19.

**Methods:** In this retrospective cohort study, we analyzed data from 28 corticosteroid-treated patients with non-severe but advanced COVID-19, in which short-course and low-dose corticosteroids were administered because of unremitting or worsening clinical conditions during hospitalization. To compare the effect of corticosteroids on viral clearance, 44 corticosteroid-untreated patients were included as controls.

**Results:** At the time of admission, corticosteroid-treated patients (*n* = 28) had a more advanced baseline illness compared with corticosteroid-untreated patients (*n* = 44), as reflected by poorer blood laboratory parameters (lymphocytes, C-reactive protein, and lactate dehydrogenase) and more extensive chest computed tomography (CT) abnormalities. Corticosteroids were given because of radiological evidence of pneumonia progression (26/28) and/or unremitting fever (22/28) after admission. The median time from illness onset to corticosteroid treatment was 9 days (IQR, 7–10). The median duration and accumulated dose of corticosteroid treatment were 4.5 days [interquartile range (IQR), 3–5] and 140 mg of methylprednisolone (IQR, 120–200). Intravenous immunoglobulin (20 g per day for 3–5 days) was co-administered with corticosteroids. With the corticosteroid treatment, all patients achieved an abatement of fever within 1 day, and 78.6% (22/28) of the patients achieved radiological remission when evaluated about 3 days later. Only one (3.6%) patient progressed to severe COVID-19, and all patients recovered and were discharged without any sequela. The median time from illness onset to viral clearance was similar, as compared with 44 corticosteroid-untreated patients with relatively milder disease [18 (IQR 14.3–23.5) days vs. 17 (IQR, 12–20) days, *p* = 0.252]. When adjusted for age, sex, underlying comorbidities, baseline blood laboratory parameters, viral load, and chest radiological findings, the causal hazard ratio of corticosteroid treatment for the viral clearance was 0.79 (95%CI, 0.48–1.30, *p* = 0.34).

**Conclusion:** Short-course and low-dose applications of corticosteroids, when co-administered with intravenous immunoglobulin, in non-severe COVID-19 patients during the stage of clinical deterioration may possibly prevent disease progression, while having a negligible impact on the viral clearance.

## Introduction

In December 2019, a pneumonia related to coronavirus disease 2019 (COVID-19) was discovered in Wuhan, China (1). The causative pathogen of this novel disease was severe acute respiratory syndrome coronavirus 2 (SARS-CoV-2) (2, 3). It is recognized that patients with coronavirus disease 2019 may have variable degrees of disease severity, ranging from asymptomatic infection to life-threatening respiratory failure (4–6). Because of its high capability of human-to-human transmission, it spread rapidly in China. At present, COVID-19 has become a major public health issue of global concern. As of June 9, 2020, there were more than 7 million confirmed cases of COVID-19, leading to over 0.4 million deaths (7). Most of the investigated antivirals, such as lopinavir/ritonavir, arbidol, chloroquine, and hydroxychloroquine, have failed to significantly improve the prognosis of COVID-19 (8–10). Preliminary reports from a randomly controlled trial showed that remdesivir could shorten the median recovery time of COVID-19 from 15 to 11 days; however, the mortality of COVID-19 by the second week was still high (7.1% in patients treated with remdesivir) (11). Moreover, for patients with severe COVID-19, a study showed that remdesivir was not associated with a better clinical outcome (12). There was no difference in clinical improvement between a 5-day course and a 10-day course of remdesivir therapy in severe COVID-19 (13). Taken together, current evidence suggests that antiviral therapy does not substantially decrease the case fatality rate of COVID-19.

Besides antiviral therapy, anti-inflammatory therapy for COVID-19 is also attracting considerable research interest. Members of a WHO panel on clinical management for COVID-19 and the Chinese Thoracic Society have conflicting opinions regarding the use of corticosteroids in COVID-19 (14, 15). While the former discourages the use of corticosteroids in COVID-19, the latter advises that corticosteroids should be administered in critically ill patients (14, 15). At the beginning of the COVID-19 outbreak in China, there was very limited knowledge of the optimal treatment for COVID-19. One of the reasons for favoring corticosteroid treatment in COVID-19 is that the use of corticosteroids in critically ill SARS patients was associated with lowered mortality and shorter hospital stays (16). Of note, when lung damage has already occurred, the case fatality rate of COVID-19 is unacceptably high, even though use of corticosteroids is not uncommon in this population (5, 6). An alternative strategy is application of corticosteroids in COVID-19 patients with clinical deterioration but before they develop severe illness. If corticosteroids could alleviate the clinical progression at this stage, then the therapy may possibly decrease the cases of severe illness and therefore lower the case fatality rate of COVID-19.

Thus far, limited data are available regarding the use of corticosteroid treatment in patients with non-severe COVID-19. In our clinical practice, short-course, and low-dose corticosteroids were administered to non-severe COVID-19 patients when there was unremitting or worsening clinical conditions. The present study retrospectively analyzed the patients' data to evaluate whether this strategy could possibly prevent disease progression and to explore the effect of the corticosteroids-based therapy on viral clearance.

## Materials and Methods

### Study Design and Population

A single-center, retrospective study was conducted at the Second Hospital of Nanjing, China. In Nanjing, the Second Hospital of Nanjing was the only designated hospital for managing patients with COVID-19. The expert panel on the management of COVID-19, in the second hospital of Nanjing, did not dissuade the use of corticosteroids providing that the treatment was closely monitored. The indication for corticosteroid treatment in the second hospital of Nanjing included severe COVID pneumonia and non-severe COVID pneumonia with evidence of disease progression. The principle of this therapy is short-course (within 1 week) and low-dose (methylprednisolone, 40 mg per day intravenously) application of corticosteroids. Intravenous immunoglobulin (20 g per day for 3–5 days) was co-administered with corticosteroids. The decision on initiation of corticosteroid treatment and duration of this medicine was made by the treating physicians. The expert panel would dynamically monitor the effects of corticosteroid treatment and had the authority to withdraw corticosteroid treatment if there was any clue that disadvantages of the treatment were outweighing the advantages.

We searched the COVID-19 database of the second hospital of Nanjing and included all the symptomatic cases fulfilling the following criteria: (1) admitted from Jan 20, 2020 to Feb 16, 2020; (2) at least 18 years of age; (3) received corticosteroid treatment because of clinical progression of COVID-19; and (4) with non-severe COVID-19 pneumonia both at the time of admission and at the time of starting corticosteroid treatment. With this strategy, 28 corticosteroid-treated patients with non-severe COVID-19 were identified (referred to as corticosteroid group). For the purpose of comparing the effect of corticosteroids on viral clearance, all 44 corticosteroid-untreated symptomatic patients, with non-severe COVID-19 pneumonia, who were at least 19 years old, and admitted during the same period were included in this study (referred to as non- corticosteroid group). None of the patients tested positive for human immunodeficiency viruses (HIV) antibody.

The diagnosis of COVID-19 was based on positive nucleic acid tests for SARS-COV-2 from a throat swab sample. The medical records, including demographic data, medical history, underlying comorbidities, symptoms, signs, laboratory parameters, radiological findings, treatments, and outcomes, were collected from the electronic health record system, and were retrospectively analyzed. This study was approved by the ethics committee of the second hospital of Nanjing (reference number: 2020-LS-ky003). Written informed consent was obtained from patients in this study.

### Laboratory Nucleic Acid Test

During inpatient days, SARS-COV-2 viral loads from throat swab specimens were evaluated every other day using quantitative reverse transcription polymerase chain reaction (qRT-PCR) kits (BGI Genomics, Beijing, China) following WHO guidelines, as previously described (4). Total nucleic acids were extracted from 200 μl virus preservation solution containing throat swabs through an automatic nucleic acid extraction system (BioPerfectus technologies company). Primers and probe sets were designed targeting open reading frame 1ab/N (forward primer 5′-AGAAGATTGGTTAGATGATGATAGT-3′; reverse primer 5′-TTCCATCTCTAATTGAGGTTGAACC-3′; and probe 5′-FAM-TCCTCACTGCCGTCTTGTTGACCA- BHQ1-3′. The human glyceraldehyde 3-phosphate dehydrogenase gene was used as an internal control (forward primer 5′-TCAAGAAGGTGGTGAAGCAGG-3′; reverse primer 5′-CAGCGTCAAAGGTGGAGGAGT- 3′; probe 5′-VIC-CCTCAAGGGCATCCTGGGCTACACT-BHQ1- 3′). The following program was run in the ABI7500 thermocycler: 50°C for 20 min; 95°C for 10 min; 40 cycles of 95°C for 15 s, 60°C for 30 s. The cycle threshold (Ct) value from the qRT-PCR reaction was used to relatively represent the viral load of SARS-COV-2.

### Radiological Assessment of Pulmonary Lesions

Chest computed tomography (CT) scans were performed every 2–4 days until a demonstration of substantial improvement of the pulmonary lesions. A semi-quantitative method was used to relatively represent the severity of pulmonary lesions, as described previously (17). Based on the extent of lung involvement, each lung lobe was visually scored from 0 to 5. No involvement was assigned a score of 0 and lobe involvements of <5, 5–25, 26–49, 50–75, and >75% were given scores of 1, 2, 3, 4, and 5, respectively. The score of each chest CT was the sum of individual lobar score, and therefore could be ranged from 0 to 25. This semi-quantitative method, however, was insensitive to evaluate the dynamic changes of pulmonary lesions. Therefore, the dynamic changes of pulmonary lesions were also evaluated qualitatively, in which the changes of pulmonary lesions could be classified as resolution, stabilization, and progression. Two experienced doctors (Chuanjun Xu and Wenkui Sun) with more than 10 years of experience in thoracic radiology reviewed the CT images blindly and determined final scores by consensus.

### Outcomes and Study Definitions

The primary outcome was progression to severe illness. Secondary outcomes were viral clearance and length of hospital stay. The case definition of severe COVID-19 pneumonia followed the Chinese interim guidance of novel coronavirus pneumonia (version 7.0). Severe cases should meet one of the follow criteria: (1) respiratory rate of 30 per min or more, (2) oxygen saturation of 93% or less while patients were breathing ambient air;, or (3) ratio of the partial pressure of arterial oxygen (Pao2) to the fraction of inspired oxygen (Fio2) ≤300 mg Hg. Also, the above-mentioned criteria should not be explained by cardiac insufficiency. Viral clearance was defined as when two-consecutive throat-swab samples obtained at least 24 h apart tested negative for SARS-CoV-2 RNA.

### Statistical Analysis

Continuous variables were expressed as the median and interquartile ranges (IQR). Categorical variables were summarized as the counts and percentages in each category. Comparison between groups was done using the Mann–Whitney *U*-test or Wilcoxon signed-rank test for continuous variables, and Chi-Square test or McNemar test for categorical variables, as appropriate. The time to viral clearance was portrayed by Kaplan–Meier plot. The above-mentioned statistical analysis was done by SPSS version 22.0 (IBM). Considering the potential selection bias in our observational data, we applied the marginal structural models and performed inverse probability weights to adjust the bias and identify the causal effect of corticosteroids usage on viral clearance. We applied R package “ipw” to accomplish inverse probability weights (18). Statistical analysis was carried out using software package R (version 3.6.3). The variables included to calculate weights contained age, sex, underlying comorbidities, and baseline characteristics including blood laboratory parameters, SARS-COV-2 viral load from throat swab sample, and pulmonary radiological findings. A *P* < 0.05 is considered statistically significant.

## Results

### Characteristics of the Patients

The median time from illness onset to admission was 5 days (IQR, 2–7), with no significant difference between corticosteroid group and non-corticosteroid group. At the time of admission, patients in the corticosteroid group were older, and had a higher percentage of patients with fever or shortness of breath, lower lymphocyte count or percentage, higher lactate dehydrogenase (LDH) level, and wider range of pulmonary involvement, compared with those in the non-corticosteroid group (*p* < 0.05, Table 1). The serum C-reactive protein (CRP) level was also higher in the corticosteroid group, although it did not reach statistical significance (*p* = 0.051). There were no obvious changes of liver and renal functions, or in the coagulation profile (Table 1). None of the patients had elevated cardiac troponin I level. The baseline viral load was comparable between the corticosteroid group and non-corticosteroid group (Table 1).

**Table 1:** Baseline demographics, clinical, laboratory, and radiological characteristics of 72 patients with COVID-19.

	**Total (*n =* 72)**	**Corticosteroids treated (*n =* 28)**	**Corticosteroids untreated (*n =* 44)**	***p*-value**
**Demographics and clinical characteristics**				
Age, years	46 (33–57)	53 (43–63)	38 (31–53)	0.004
>60	16 (22.2%)	10 (35.7%)	6 (13.6%)	0.028
Sex				0.084
Female	32 (44.4%)	16 (57.1%)	16 (36.4%)	
Male	40 (55.6%)	12 (42.9%)	28 (63.6%)	
Currently smoking	6 (8.3%)	2 (7.1%)	4 (9.1%)	1.000
Comorbidity	15 (20.8%)	7 (25.0%)	8 (18.2%)	0.487
Hypertension	10 (13.9%)	4 (14.3%)	6 (13.6%)	1.000
Diabetes	4 (5.6%)	1 (3.6%)	3 (6.8%)	0.953
Other	5 (6.9%)	4 (14.3%)	1 (2.3%)	0.139
Respiratory rate >24 breaths per min	1 (1.4%)	1 (3.6%)	0 (0%)	0.389
Fever (temperature ≥37.3°C)	51 (70.8%)	24 (85.7%)	27 (61.4%)	0.027
Cough	57 (79.2%)	24 (85.7%)	33 (75.0%)	0.275
Shortness of breath	19 (26.4%)	13 (46.4%)	6 (13.6%)	0.002
Time from illness onset to admission, days	5 (2–7)	5.0 (2.0–7.0)	3.5 (2.0–7.0)	0.459
**Blood laboratory findings**				
White blood cell count, × 10^9^ /L	4.31 (3.45–4.79)	4.02 (3.47–5.08)	4.33 (3.45–4.79)	0.831
<4	32 (44.4%)	14 (50.0%)	18 (40.9%)	0.449
Lymphocyte count, × 10^9^ /L	1.18 (0.94–1.54)	1.00 (0.74–1.41)	1.36 (1.07–1.65)	0.006
<0·8	13 (18.1%)	10 (35.7%)	3 (6.8%)	0.002
Hemoglobin, g/L	136 (127–150)	129 (122–137)	142 (130–154)	0.001
<120	9 (12.5%)	5 (17.9%)	4 (9.1%)	0.465
Platelet count, × 10^9^ per L	172 (146–218)	167 (146–215)	184 (144–219)	0.575
<100	4 (5.6%)	3 (10.7%)	1 (2.3%)	0.319
Albumin, g/L	44.3 (42. 0–47.6)	43.8 (41.9–45.6)	44.5 (42.1–48.6)	0.154
<35	2 (2.8%)	2 (7.1%)	0 (0%)	0.148
Alanine aminotransferase, IU/L	21.8 (14.6–33.2)	21.3 (13.4–31.0)	22.0 (15.6–35.0)	0.885
>40	12 (16.7%)	5 (17.9%)	7 (15.9%)	1.000
eGFR	115.8 (103.0–127.3)	113.6 (101.0–123.2)	118.4(105.9–128.1)	0.225
<90	4 (5.6%)	2 (7.1%)	2 (4.5%)	1.000
Lactate dehydrogenase, IU/L	203 (156–250)	226 (194–303)	182 (150.0–213)	0.004
>245	19 (26.4%)	11 (39.3%)	8 (18.2%)	0.048
Creatine kinase, U/L	57 (41–103)	68 (40–130)	56(41–82)	0.196
>140	10 (13.9%)	6 (21.4%)	4 (9.1%)	0.260
Cardiac troponin I, ng/mL	0.05 (0.13–0.09)	0.05 (0.02-0.09)	0.05 (0.01–0.1)	0.954
Prothrombin time, s	12.1 (11.8–12.9)	12.3 (11.8–13.0)	12.0 (11.6–12.7)	0.509
>14	9 (12.5%)	4 (14.3%)	5 (11.4%)	1.000
C-reactive protein, mg/l	8.0 (2.7–21.3)	10.7 (4.3–33.1)	5.6 (1.5–17)	0.051
>10	29 (40.3%)	15 (53.6%)	14 (31.8%)	0.067
D-dimer, μg/L	0.23 (0.16–0.34)	0.19 (0.18–0.34)	0.25 (0.15–0.37)	0.861
>0.55	4/67 (6.0%)	1/25 (4.0%)	3/42 (7.0%)	1.000
Procalcitonin, ng/mL	0.02 (0.01–0.04)	0.02 (0.01–0.04)	0.02 (0.01–0.06)	0.729
> 0.1	10 (13.9%)	3 (10.7%)	7 (15.9%)	0.786
**Microbiological data**				
Viral load (Ct value)^a^	30 (25.3–34.0)	30 (25.3–33.8)	30 (25.3–34)	0.746
**Radiological features**				
Bilateral pulmonary infiltration	48 (66.7%)	21 (75%)	27 (61.4%)	0.231
Multiple lobes involvement	52 (72.2%)	22 (78.6%)	30 (68.2%)	0.337
All lobes involvement	19 (26.4%)	13 (46.4%)	6 (13.6%)	0.002
CT score	4 (2–6.8)	5.5 (3.3–8.0)	3.5 (2–5.8)	0.023

*Data were expressed as median (IQR), n (%), n/N(%). Comparison between groups was done using Mann-Whitney U-test, Chi-Square test, or Fisher's exact test, as appropriate*.

a *the cycle threshold (ct) value from quantitative reverse transcription polymerase chain reaction was used to relatively represent the viral load of SARS-COV-2*.

### Treatment

All the patients received aerosol interferon-alpha during their stay in hospital. Oral antiviral drugs, such as arbidol and HIV protease inhibitors (lopinavir/ritonavir or darunavir/cobicistat), were administered to the majority of the patients (Table 2). Use of antibiotics was seen in 39.3% (11/28) and 9.1% (4/44) of the patients in the corticosteroid group and non-corticosteroid group, respectively (*p* = 0.002). However, none of the 15 antibiotic-treated patients had a confirmed bacterial infection.

**Table 2:** Treatments and outcomes for 72 patients with COVID-19.

	**Total (*n =* 72)**	**Corticosteroids treated (*n =* 28)**	**Corticosteroids untreated (*n =* 44)**	***p*-value**
**Treatments**				
Aerosol interferon-alpha	72 (100%)	28 (100%)	44 (100%)	…
Arbidol	37 (51.4%)	15 (53.6%)	22 (50.0%)	0.768
Lopinavir/ritonavir or darunavir/cobicistat	66 (91.7%)	27 (96.4%)	39 (88.6%)	0.466
Intravenous immunoglobulin	40 (55.5%)	28 (100%)	12 (27.3%)	<0.001
Antibiotics	15 (20.8%)	11 (39.3%)	4 (9.1%)	0.002
High-flow nasal cannula oxygen therapy	0 (0%)	0 (0%)	0 (0%)	…
Mechanical ventilation	0 (0%)	0 (0%)	0 (0%)	…
**Outcomes**				
Progressed to severe illness	1 (1.4%)	1 (3.6%)	0 (%)	0.389
Time from illness onset to viral clearance, days	17.5 (12.3–21)	18 (14.3–23.5)	17 (12–20)	0.252
Hospital stay, days	20.0 (12.0–27.8)	25 (16.3–30.0)	14.5 (10–26)	0.016
>21 days	32 (44.4%)	19 (67.9%)	13 (29.5%)	0.001

*Data were expressed as median (IQR) or n (%). Comparison between groups was done using Mann-Whitney U test, Chi-Square test, or Fisher's exact test, as appropriate*.

Of the 28 patients in the corticosteroid group, 26 had follow-up chest CT results before the corticosteroid treatment, all of which demonstrated pneumonia progression and 76.9% of which had fever (peak body temperature ranged from 37.5 to 39.0°C). The median CT score increased from 5.5 (IQR, 3.3–8.0) to 8.0 (IQR, 5.3–11) (*p* < 0.001, Table 3). Two patients did not have follow-up chest CT scans before corticosteroid administration to evaluate whether there was radiological progression of pneumonia. Corticosteroids were given to these two patients due to unremitting fever after admission. There was a significant increase of CRP at the time of corticosteroid administration as compared with baseline (*p* = 0.007, Table 3).

**Table 3:** Changes of laboratory and radiological characteristics in 28 corticosteroid-treated patients.

	**Patients with paired data, No**.	**At the time of admission**	**At the time of initiation of corticosteroids**	***p*-value**
**Blood laboratory fingdings**				
White blood cell count, × 10^9^ /L	27	4.06 (3.57–5.19)	4.14 (3.43–5.04)	0.286
<4	27	13/27 (48.1%)	13/27 (48.1%)	1.000
Lymphocyte count, × 10^9^ /L	27	1.01 (0.74–1.43)	0.97 (0.64–1.28)	0.523
<0·8	27	10 (37.0%)	11 (40.7%)	1.000
Lactate dehydrogenase, IU/L	26	226 (189–291)	233 (223–337)	0.134
>245	26	10 (38.4%)	10 (38.4%)	1.000
C-reactive protein, mg/l	26	10.7 (4.4–36.6)	18.8 (9.7–51.7)	0.007
>10	26	12 (46.1%)	19 (73.1)	0.063
**Microbiological data**				
Viral load (Ct value)^a^	28	30 (25.3–33.8)	31.5 (28.0–35.0)	0.082
Virus clearance	28	0 (0%)	2 (7.1%)	0.500
**Radiological features**				
Bilateral pulmonary infiltration	26	19 (73.1%)	25 (96.2%)	0.031
Multiple lobes involvement	26	20(76.9%)	26 (100.0%)	0.013
All lobes involvement	26	12 (46.2%)	13 (50.0%)	1.000
CT score	26	5.5 (3.3–8.0)	8.0 (5.3–11)	<0.001

*Data were expressed as median (IQR) or n (%). Comparison between groups was done using Wilcoxon signed-rank test or McNemar test, as appropriate*.

a *the cycle threshold (ct) value from quantitative reverse transcription polymerase chain reaction was used to relatively represent the viral load of SARS-COV-2*.

The median time from illness onset to corticosteroid treatment was 9 days (IQR, 7–10). At the time of starting corticosteroid treatment, the median respiratory rate was 20 breaths per min (IQR, 20–21). The median duration and accumulated dose of corticosteroid treatment were 4.5 days (IQR, 3–5) and 140 mg of methylprednisolone (IQR, 120–200). The maximum duration and accumulated dose of corticosteroid treatment were 10 days and 400 mg of methylprednisolone.

### Outcomes

**Figure 1:**
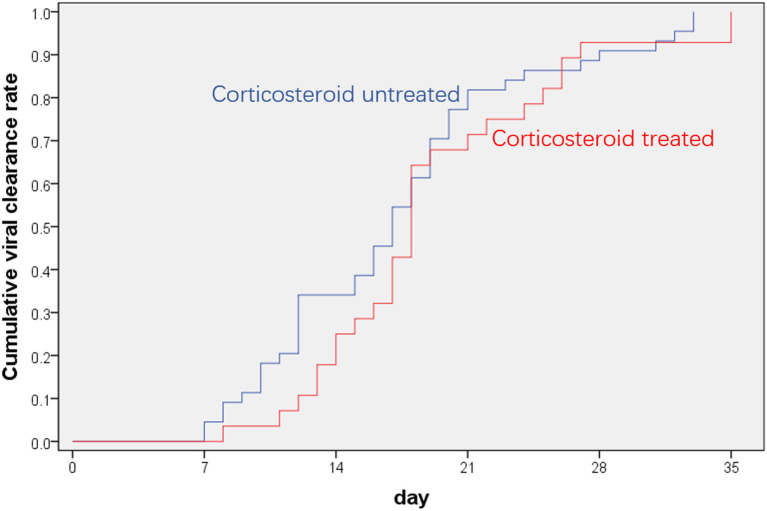
Time to viral clearance in patients with COVID-19.

All the patients in the corticosteroid group and non-corticosteroid group recovered and were discharged from the hospital without apparent sequela. One (3.6%) patient in the corticosteroid group progressed to severe COVID-19 at the time of corticosteroid discontinuation (Table 2, Supplemental Figure 1). The time from illness onset to viral clearance in the corticosteroid group did not differ significantly from that in the non-corticosteroid group [18 (IQR 14.3–23.5) days vs. 17 (IQR,12–20) days, *p* = 0.252] (Table 2, Figure 1). After adjusting for age of more than 60 years, sex, any underlying disease, and baseline parameters (LDH of more than 245 IU/L, lymphocytes of <0.8 × 10^9^ cells/L, CRP of more than 10 mg/L, viral load [Ct value] and number of lung lobe involvement), the causal hazard ratio of corticosteroids for viral clearance was 0.79 (95%CI, 0.48–1.30, *p* = 0.34). Of note, patients in the corticosteroid group had a significantly longer length of hospital stay as compared with those in the non-corticosteroid group [25 days (IQR,16.3–30) vs. 14.5 days [10–26], respectively; *p* = 0.016, Table 2].

At the time of starting corticosteroid treatment, 78.6% (22/28) of the patients had fever. The median axillary temperature was 38.2°C (IQR, 37.9–38.7). With corticosteroid treatment, all patients achieved an abatement of fever within 1 day. However, four patients had transient fever (<24 h) after corticosteroid discontinuation. When the severity of pneumonia was evaluated qualitatively, chest CT performed 2–4 days after starting corticosteroid treatment showed resolution of pneumonia in 78.6% (22/28) and progression of pneumonia in 22.4% (6/28) of the patients. For the six patients with continuing progression of pneumonia after 2–4 days of corticosteroid treatment, follow-up chest CT obtained about 3 days later showed resolution of pneumonia in five patients and stabilization of pneumonia in one patient. When the severity of pneumonia was evaluated with the semi-quantitative method, the median CT score decreased from 8 (IQR, 5.3–11) before/at the time of corticosteroid therapy to 6 (IQR, 5.0–8.7) about 1 week after imitation of corticosteroid treatment (*p* = 0.001). Also, CRP level decreased from 15.5 (IQR, 9.6–51.3) to 4.6 (IQR, 1.7–10.4) (analyzed on 24 paired results, *p* = 0.007). Finally, the fasting blood glucose level did not significantly change [before corticosteroid treatment, 5.1(IQR, 4.3–5.9); 1 week after starting corticosteroid treatment, 4.42 (IQR, 4.2–6.7); *p* = 0.845].

## Discussion

The pathogenesis of COVID-19 is still not well-understood. One of the unresolved issues is which factor, of “direct injury by virus” and “immune damage triggered by virus,” contributes more to the lung destruction. Accumulating evidence suggests that the severity of COVID-19 correlates with a hyper-inflammatory status resembling a cytokine storm (19–21). Autopsy findings in a patient with severe COVID-19 revealed interstitial mononuclear inflammatory infiltrates in both lungs. Although there was profound lymphocytopenia in peripheral blood, the CD4 and CD8 T lymphocytes were hyperactivated (22). Based on those findings, immunomodulatory drugs, including corticosteroids, were advocated for in severe COVID-19 by some experts (23, 24). Nevertheless, when severe illness has already occurred, the management of COVID-19 could be more difficult, requiring more extensive medical interventions. At this stage of severe illness, patients may be more vulnerable to corticosteroid-related side effects. If aberrant immune responses lead to worsening of COVID-19, it is reasonable to use corticosteroids during clinical deterioration, preferably before the stage of severe illness.

In our study, 28 patients with non-severe COVID-19 were given short-course and low-dose corticosteroids because of continuing clinical progression or unresolved illness during hospitalization (Table 3). The responses to corticosteroids were favorable, with rapid abatement of fever within 1 day. Only one (3.6%) corticosteroid-treated patient progressed to severe illness (specifically, after corticosteroid discontinuation), although we also found substantial improvement of pulmonary lesions during corticosteroid treatment in this patient (Supplemental Figure 1). None of the 28 corticosteroid-treated patients required high-flow nasal cannula oxygen therapy or mechanical ventilation. All patients recovered and were discharged from the hospital. The findings suggested short-course and low-dose application of corticosteroids may alleviate the clinical progression of COVID-19. When this strategy is applied to the non-severe cases during the stage of clinical deterioration, the proportion of patients progressing to severe illness may be decreased. Of note, the time of initiating corticosteroid treatment was 9 days (IQR, 7–10) from illness onset. Closely monitoring the patients should be done around this time point, as there may be rapid acceleration of COVID-19.

Immunosuppression therapy for COVID-19 always raised concerns about the impairment of viral clearance (14, 15, 25). In patients with severe acute respiratory syndrome and Middle East respiratory syndrome, corticosteroid treatment was associated with slower viral elimination (26, 27). In our study, the duration of viral clearance in the corticosteroid group did not differ significantly from that in the non-corticosteroid group (Table 2). After adjusting for confounding factors, the causal hazard ratio of corticosteroids on viral clearance was 0.79 (95%CI, 0.48–1.30, *p* = 0.34). Nevertheless, it is still less understood to what extent the time to viral clearance influences the survivor. In a group of asymptomatic infections, the patients had mild lung damage or even had normal chest CT despite the fact that many of them could not clear the virus quickly (4). Asymptomatic infections may be an example of viral adaptation to host immune responses.

In our study, corticosteroid-treated patients had more advanced COVID-19 compared with corticosteroid-untreated patients, as reflected by poorer blood laboratory parameters (lymphocytes, CRP, and LDH) and more extensive chest CT involvement (Table 1). This may partially explain why corticosteroid-treated patients had longer hospital stays. Four of the 28 corticosteroid-treated patients had transient fever (<24 h) after corticosteroids were discontinued. It was unclear whether it was related to a secondary infection or a residual abnormal immune reaction to the virus. Finally, we did not observe a significant impact of short-course and low-dose application of corticosteroids on the fasting blood sugar level.

This study was limited by a relatively small sample size that may not have the statistical power to adjust the confounding prognostic factors contributing to viral clearance. Despite the method we used to do causal inference, our analysis was based on observational data, and there would still be some biases that cannot be adjusted for. Additionally, due to the retrospective design and lack of comparable controls with similar disease severity, it was difficult to draw firm conclusions regarding the ability of corticosteroid treatment to prevent COVID-19 from progressing to severe illness. A larger scale cohort study or random controlled trial could help to further assess the role of corticosteroids on the prognosis of COVID-19. Finally, as intravenous immunoglobulin was co-administered with corticosteroids in our study, it was suggested that the intervention should be recognized as a combination of corticosteroids and intravenous immunoglobulin.

In conclusion, short-course and low-dose administration of corticosteroids (combined with intravenous immunoglobulin) in non-severe COVID-19 during the stage of clinical deterioration may possibly prevent disease progression and reduce the risk of the disease developing into severe illness. This strategy may not significantly impact the viral clearance. The findings in our study would encourage the carrying out of larger cohort studies or randomly controlled trials to further evaluate the role of corticosteroid treatment on COVID-19.

## Data Availability Statement

The datasets used and/or analyzed during the current study are available from the corresponding author on reasonable request.

## Ethics Statement

The studies involving human participants were reviewed and approved by the ethics committee of the second hospital of Nanjing (reference number: 2020-LS-ky003). The patients/participants provided their written informed consent to participate in this study.

## Author Contributions

ZH, CCheng, YC, HW, CH, YZ, XZ, and YY were the members of the expert panel for the management of COVID-19 in the second hospital of Nanjing. They designed the study and actively monitored the patients. ZH, XZ, and YY analyzed the data. ZH, YL, CX, and WS wrote the manuscript. CX and WS evaluated the radiological features. WC performed the nucleic acid test. CChen and DJ collected and analyzed the data. ZP and XC performed the statistical analysis. All authors contributed to the article and approved the submitted version.

## Conflict of Interest

The authors declare that the research was conducted in the absence of any commercial or financial relationships that could be construed as a potential conflict of interest.

## Abbreviations

COVID-19: coronavirus disease 2019
SARS-CoV-2: severe acute respiratory syndrome coronavirus 2
HIV: human immunodeficiency viruses
qRT-PCR: quantitative reverse transcription polymerase chain reaction
CT: computed tomography
Pao2: partial pressure of arterial oxygen
Fio2: fraction of inspired oxygen
IQR: interquartile ranges
LDH: lactate dehydrogenase
CRP: serum C-reactive protein.
