# Supplementary figures and images for: Clinical Use of Short-Course and Low-Dose Corticosteroids in Patients With Non-severe COVID-19 During Pneumonia Progression
Source: Front Public Health. 2020 Jul 3;8:355. doi: 10.3389/fpubh.2020.00355 (PMC7349005; doi:10.3389/fpubh.2020.00355)

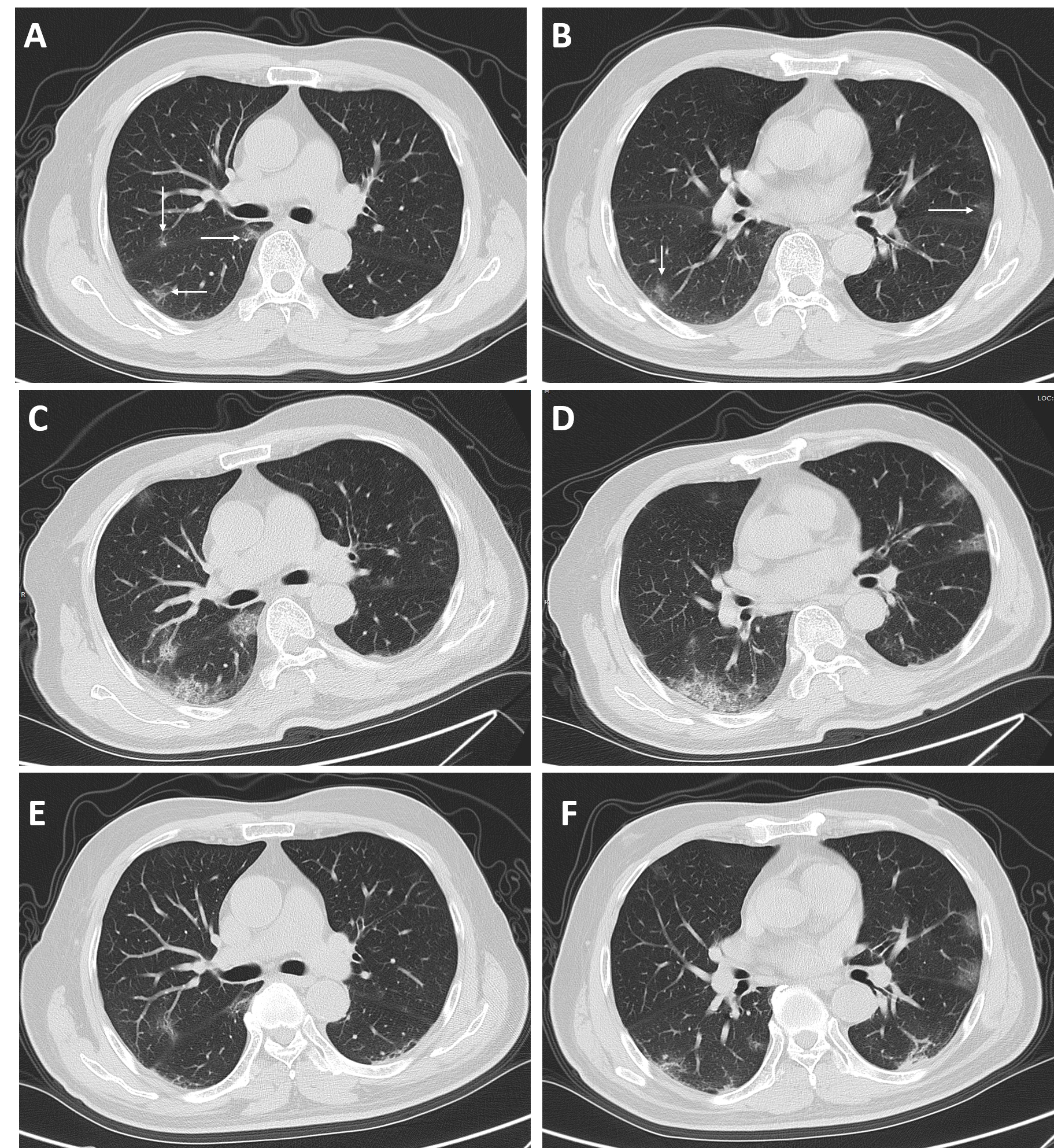

Supplement: Supplementary file 1 [file Image_1.JPEG]
